# Advances in Radiotherapy Management of Esophageal Cancer

**DOI:** 10.3390/jcm5100091

**Published:** 2016-10-21

**Authors:** Vivek Verma, Amy C. Moreno, Steven H. Lin

**Affiliations:** 1 Department of Radiation Oncology, University of Nebraska Medical Center, Omaha, NE 68198, USA; vivek.verma@unmc.edu; 2 Department of Radiation Oncology, University of Texas M.D. Anderson Cancer Center, Houston, TX 77030, USA; akmoreno@mdanderson.org

**Keywords:** esophageal cancer, three-dimensional conformal radiation therapy, intensity-modulated radiation therapy, proton beam therapy, trimodality therapy

## Abstract

Radiation therapy (RT) as part of multidisciplinary oncologic care has been marked by profound advancements over the past decades. As part of multimodality therapy for esophageal cancer (EC), a prime goal of RT is to minimize not only treatment toxicities, but also postoperative complications and hospitalizations. Herein, discussion commences with the historical approaches to treating EC, including seminal trials supporting multimodality therapy. Subsequently, the impact of RT techniques, including three-dimensional conformal RT, intensity-modulated RT, and proton beam therapy, is examined through available data. We further discuss existing data and the potential for further development in the future, with an appraisal of the future outlook of technological advancements of RT for EC.

## 1. Introduction

Esophageal cancer (EC) is a major health burden both in the USA and abroad; it is the eighth most common cancer worldwide and is associated with poor prognosis, producing over 400,000 deaths yearly [1]. Owing to the close anatomical apposition of the esophagus with several vital structures such as the heart and lung, treatment—regardless of modality—risks injuring these organs. Due to these anatomical considerations, a prime goal of RT has been to improve the safety of treatment while minimizing cardiopulmonary toxicities and/or surgical complications. This review focuses on radiation therapy (RT) as part of EC treatment. To this extent, this review emphasizes advances in RT techniques and modalities in efforts to improve toxicities. This is preceded by a discussion of historical treatment for EC, and culminates with a discussion of further potential advancements in the future.

## 2. Historical Perspective

For over a century, surgical resection was the key modality for the treatment of EC [2]. However, the earliest outcomes with surgery were poor (even by previous standards), with high rates of postoperative complications and increased propensity for both local and distant failure [2,3,4,5]. As clinical experience accumulated with surgery, refinement of techniques improved complication rates [3,4], although recurrence rates remained high. 

As a way to improve local control (LC) after surgery [6], administration of postoperative radiotherapy began. Though data demonstrated improved LC, patterns of failure shifted towards distant metastasis as the predominant mode of relapse, although local failures were still not uncommon. The rise of radiosensitizing chemotherapy revolutionized oncologic care for EC [7]. A randomized trial of nonoperative EC treatment examining combined chemoradiotherapy (CRT) versus RT alone was aborted early owing to a substantial overall survival (OS) benefit with the former [8].

There have been a number of randomized studies comparing surgical resection with or without preoperative CRT (Table 1). A study from Ireland randomized 113 patients, demonstrating a median OS improvement from 11 months in the surgery-only group to 16 months in the combined modality group [9]. Another study from the University of Michigan was not able to detect an OS difference between groups in 100 patients, reporting a median OS of 18 months in the surgery-only arm and 17 months in the trimodality arm [10]. The Trans-Tasman Radiation Oncology Group (TROG) randomized 256 patients but failed to find a progression-free survival or OS improvement with neoadjuvant therapy [11]. The CALGB 9781 trial was prematurely closed owing to poor accrual, enrolling 56 patients [12]. Despite this, outcomes overwhelmingly favored the trimodality group, with 4.5 years versus 1.8 years. This trial was notable for encompassing the most up-to-date surgical techniques and reported the lowest postoperative hospital stay and complication rate of any study to date. However, despite a meta-analysis also displaying improved outcomes with CRT over surgery alone [13], owing to conflicting results from prior data, the issue remained unresolved until the Chemoradiotherapy for Oesophageal Cancer Followed by Surgery Study (CROSS) trial, which cemented the use of the trimodality treatment [14]. This large, 366-patient randomized Dutch trial compared trimodality therapy to surgery alone. This trial was a departure from several trials in the past. In addition to the large sample size, most patients had adenocarcinoma, anatomically lower tumors, underwent a distinct chemotherapy regimen (carboplatin/paclitaxel), as well as a lower RT dose (41.4 Gy). Accordingly, results were also starkly distinct from prior randomized trials, including a doubling of OS in the trimodality group, with 48.6 months vs. 24.0 months. What was striking as well was that the relative survival benefit of trimodality therapy was larger for squamous cell carcinoma (81.6 months vs. 21.1 months, *p* = 0.008) than adenocarcinoma (43.2 months vs. 27.1 months, *p* = 0.038). There was also no observed increase in perioperative complications from neoadjuvant CRT, as postoperative death in the hospital or within 30 days was comparable to modern figures as observed today. These results are also noteworthy in that the alteration of chemotherapy and decreasing the RT dose drastically decreased complication rates and enhanced outcomes. Hence, in a sense, the optimal RT dose has come under question (which is important in light of using advanced techniques for the goals of dose escalation, discussed in a subsequent section). The use of new chemotherapy compounds different from decades of historical data is also a stark departure from the past.

The success of combined CRT prompted investigations of whether surgical resection is needed after CRT. Though three prospective randomized trials have not demonstrated an OS benefit to esophagectomy, other endpoints have been relevant (Table 2). A study from Hong Kong, randomizing between CRT and surgery, demonstrated no differences in OS between groups [17]. However, though patients were treated in the 2000s, operative mortality was 7% and postoperative complications (of various types) occurred in 39% of the cohort. Notably, the trial was also underpowered (*n* = 80). Next, a trial from Germany randomized 172 patients to CRT with or without surgery [18]. LC and progression-free survival favored the surgery arm, while treatment-related mortality was nearly four-fold higher in the trimodality group (13%). These results may be compared to those of a French randomized study with the same fundamental study design, with the exception that those patients that had a response to CRT were randomized, and the study initially allowed split-course RT in one of the arms, which was later abandoned [19]. The trimodality group experienced fewer local failures, but experienced no differences in OS. Echoing other results, this group experienced a three-month mortality of 10% and an increased in-hospital stay.

Since radiotherapy is an important component in the management of EC, the delivery of radiotherapy is an important consideration given the critical organs of the heart and lung that surround tumors in the mid to distal esophagus [20]. Radiotherapy in this area delivers a significant dose to the surrounding organs, and therefore advanced techniques to reduce the dose exposure of these organs will mitigate the toxicities and improve outcomes for patients. We review the evidence that advanced radiotherapy delivery is a critical component in improving treatment outcomes in esophageal cancer. 

## 3. Intensity-Modulated Radiotherapy

Radiation therapy has rapidly advanced over the past several decades, coincident with technological advances. Historically, RT was planned and delivered in a two-dimensional manner, which relied on external anatomical landmarks to localize a general area where to deliver RT. The development of computed tomography (CT) scans to create a “virtual 3D patient” on which to perform RT treatment planning led to the technique known as three-dimensional conformal radiotherapy (3DCRT). As its name suggests, the three-dimensional nature of tumor localization, made possible by CT imaging, offers more precise target localization than two-dimensional RT. Many of the seminal trials in EC have utilized 3DCRT, including the CROSS trial; this is summarized in Table 2 along with other data [15,16]. As a result, 3DCRT remains the current standard RT technique for EC. However, the evolution of technology has resulted in the construction of computer algorithms that can modify individual RT beam profiles so as to create highly conformal RT dose distributions. Input to these algorithms includes various dose constraints to organs known to be at risk of RT toxicities (e.g., lung, heart, spinal cord). Based on prioritizing dose limitations to these organs at risk, this so-called “inverse planning” algorithm can create non-uniform fluence patterns to each of a number of complex beam arrangements. The result is highly conformal RT delivery, more so than that of 3DCRT, which is termed intensity-modulated radiation therapy (IMRT). The ability to tailor the treatment plan by setting dose constraints to organs at risk is the largest advantage of IMRT; 3DCRT rather uses “forward-planning” and involves manually modifying beam profiles and then examining doses to organs at risk, instead of vice-versa. Though IMRT is more labor-intensive, the greater number of beams may result in more low-dose regions and subsequently decrease cardiopulmonary doses [21,22] while maintaining appropriate tumor doses. Moreover, in patients with comorbidities, IMRT may be able to constrain the heart and lungs to specific predetermined levels, thus potentially modifying the dose to sensitive organs based on the individual risk of injury. The importance of these notions is that doses to these organs at risk correlate with postoperative complications [23,24,25] and potentially even outcomes [26]. For instance, though wound complications are no different between groups (10% 3DCRT vs. 11% IMRT (*p* = 0.36)), pulmonary complications are increased with 3DCRT (30% vs. 24%, *p* = 0.02). 

To date, however, aside from a small, 60-patient randomized study that showed no statistical differences in outcomes [27], there have been no randomized studies examining outcomes in patients treated with 3DCRT-based CRT compared to that using IMRT. Table 3 summarizes retrospective, largely single-institutional data of IMRT-based treatment for locally advanced EC [28,29,30,31]. The largest study to date from MD Anderson Cancer Center has examined 676 patients, 413 of which were treated with 3DCRT and 263 IMRT [26]. Using multivariate analyses of the inverse probability of treatment weighting (IPTW) methodology, and despite the IMRT group being associated with worse performance status, 3DCRT modality was found to be an independent predictor of all-cause mortality. Though causes of mortality were often undocumented, cardiac-specific mortality was also statistically increased in the 3DCRT group. Further analysis determined that locoregional control was superior in the IMRT group but not distant metastatic-free survival or cancer-specific survival. 

These data have also been used to examine perioperative complications with 3DCRT versus IMRT. Wang et al. evaluated 30-day postoperative complications experienced by 444 patients treated with neoadjuvant CRT followed by surgery [26]. As compared to the 208 patients treated with 3DCRT, fewer postsurgical gastrointestinal and pulmonary complications were experienced in the 164 and 72 patients treated with IMRT and proton beam therapy (PBT), respectively. On multivariate analysis, dose-volume parameters such as mean lung dose were most correlated with postoperative complications. This has been shown to be a strong advantage of IMRT, which allows for decreased doses to organs at risk, despite the lack of randomized evidence. Nevertheless, these results have provided the strongest evidence to date that IMRT may substantially impact outcomes by means of reducing toxicities that may in turn manifest either perioperatively or during follow-up.

Further support for IMRT in reducing toxicities over 3DCRT comes from an analysis of two large cancer registries, encompassing over 2500 elderly patients [32]. Of note, IMRT had increased over 10-fold from 2002 to 2009, with a corresponding decrease in 3DCRT. On the propensity score of IPTW-adjusted multivariate analysis, adjusting for potential confounders, IMRT did not confer an independent association with pulmonary mortality. However, it was associated with decreased all-cause, other-cause, and cardiovascular mortality. Though not able to be ascertained using billing codes, it may be possible that distal lesions, occurring in greater anatomic proximity with the heart, may be most optimal for IMRT in order to decrease cardiac mortality. Nevertheless, in the absence of prospective work, these population-based data corroborate high-volume, single-institutional results in support of the clinical utility of IMRT in EC.

## 4. Proton Beam Therapy

Unlike photons (X-rays), proton beam therapy (PBT) exploits physical properties of charged particles [33]. The proton is a heavy particle that is over 1800 times heavier than an electron. As a result, its interaction with matter is substantially different than that of photons or electrons. When accelerated to mega-electron volts of energy, protons initially traverse matter with minimal loss in energy (attenuation), resulting in lower patient doses proximal to the target of interest. As protons decelerate, energy is selectively deposited in the area where protons have minimal or essentially no velocity. This peak of energy deposition is termed the Bragg peak. Beyond this point there is essentially no dose deposition. Ramifications of this physical phenomenon are that the Bragg peak can be “placed” within a target of interest, providing the full prescribed dose within the target, with virtually no dose for the beam to exit the patient. This substantially reduces the “low dose bath” which is a key advantage of PBT over techniques such as IMRT [34]. 

PBT as part of oncologic care has evolved rapidly; it is estimated that over 90 facilities will be operational worldwide by 2020, compared with the 61 facilities currently active [35]. The exorbitant cost of building the infrastructure to house such technologies, generally on the orders of hundreds of millions of dollars, brings on the central question of whether it is better over photon-based technologies, including IMRT. The central dilemma facing PBT utilization is the fact while dosimetric data supports the superiority of PBT, clinical data—especially prospective data—is greatly lacking. It is unknown whether the lower integral dose to organs at risk translates to a clinically meaningful reduction in toxicities. Central nervous system (CNS) and pediatric malignancies were among the first sites that began testing this notion in a prospective manner. Nevertheless, EC is a prime example of the type of neoplasm for which a consistent reduction in organ-at-risk doses may translate into clinical advantages [36]. To support this possibility is the evidence from many comparative dosimetric studies that have demonstrated a significant reduction in cardiopulmonary doses over IMRT and 3DCRT [37,38,39]. The close anatomical relationship between the esophagus, lungs, and heart, together with an operative procedure in the chest after CRT, elicits the belief that decreasing doses to surrounding areas may prove clinically advantageous in terms of overall toxicities and postoperative complications [40].

Clinical results of PBT for esophageal cancer are limited to two series from Japan and the MD Anderson Cancer Center (Table 4). Investigators at the University of Tsukuba initially reported a series of 51 patients, most of whom were treated with combinations of photons and PBT [41]. The median survival was 20 months, and though no chemotherapy was utilized, the ability of PBT to dose-escalate gross disease (80 GyE in this study) was evident. The group published a follow-up series with concurrent CRT [42] in 40 patients treated with cisplatin/5-fluorouracil and PBT (60 GyE). Whereas the three-year OS was 70% with a two-year locoregional control of 66%, there were importantly no grade 3 or higher toxicities. The initial MD Anderson study of CRT was reported in 2012 [43]. Unlike the Japanese cohort, these 62 patients were largely adenocarcinomas treated with various chemotherapy regimens and PBT (50.4 GyE), with a subset receiving pre-CRT induction chemotherapy. Of the entire cohort, the grade 3 toxicity rate was under 10%. Forty-seven percent underwent post-CRT surgical resection, which resulted in a pathologic complete response rate of 28%. Notably, postoperative wound, cardiac, and pulmonary complications occurred in just 3%, 8%, and 7%, respectively; the three-year OS was 52%. A further analysis of postoperative complications in a retrospective comparison with IMRT and 3DCRT has been described in the previous section [26]. Of note, the 72 patients who received PBT (versus the 164 and 208 patients receiving IMRT and 3DCRT, respectively) displayed a strong trend towards fewer postoperative pulmonary complications. On multivariate analysis, whereas the RT modality predicted for postoperative pulmonary complications, this was not true when the mean lung dose was factored in. This indicated that PBT in itself may not be associated with fewer pulmonary complications, but its primary effect (e.g., decreasing mean lung dose) is indeed associated. It is true that any modality which acts to minimize these dose parameters is comparably effective, but PBT offers the greatest dosimetric advantage to do so, along with IMRT to a lesser extent.

Lastly, though PBT is the most abundant form of heavy particle therapy used in the world, other forms have been tested for esophageal cancer. The effect of carbon ion RT was prospectively evaluated in a study from Japan [44]. Thirty-one patients were treated with a dose up to 36.8 GyE, followed by surgery. The authors reported no late toxicities and one acute toxicity, with 39% achieving a pathologic complete response. Owing to the limited availability of this form of heavy particle therapy, further data are needed.

Despite the promising aforementioned retrospective data, there are many concerns over PBT that should not be ignored. First, performing routine treatment of various neoplasms without corresponding clinical evidence (in some cases, even retrospective data) may contribute to its controversy. Second, protons are very sensitive to changes in density, and so if there was a slight change in the patient’s body positioning day to day, or a large change in the tumor mass during treatment, there would be a notable risk of marginally missing the target volume. The gastro-esophageal junction (with the diaphragm in close proximity) is such an area, and as with IMRT, patients must be planned in a manner that locates anatomic areas as a function of the breathing phase. Hence, prior to performing CT-based treatment planning, multiple sets of CT scans must be obtained, corresponding to the tumor location during various phases of breathing; this is known as four-dimensional CT acquisition. Next, because the Bragg peak needs to be spread out and placed within the target volume, fluctuations in heterodensities and/or thickness of matter may lead to under-dosing the tumor volume and/or overdosing areas proximal or distal to this volume. For instance, the presence of large gastric distension or decreased body thickness from low oral intake may lead to uncertainties in dose deposition and potentially could compromise the dose to the tumor target. In order to partially compensate for these issues, an additional safety margin is often added for PBT treatments. Another controversy is the cost; PBT centers are associated with significant installation, operational, and maintenance costs. It has been debated whether the potential, yet largely unproven, benefits of PBT can offset its high costs. Because cost-effectiveness is largely related to whether toxicity reductions can be proven, a lack of clinically documented toxicity reductions (especially in a prospective manner) also hampers true assessments of the cost-effectiveness of PBT [45,46]. However, there is good reason to believe that PBT could be cost-effective for EC, based on the aforementioned retrospective data which showed decreased clinical toxicities, postoperative complications, and hospital stay. 

## 5. Future Outlook

The rapidity with which advances in RT are occurring not only for EC but also for many other neoplasms necessitates a greater volume of high-quality experiences in order to further characterize the safety, efficacy, and justification for further use in the future. There are currently prospective trials underway that will greatly assist in further delineating the role of PBT for EC. A phase II trial being conducted at Loma Linda Medical Center is aiming to evaluate outcomes in a targeted population of 38 resectable patients undergoing carboplatin/paclitaxel and PBT (NCT01684904) [47]. The randomized phase IIB trial being led by the MD Anderson Cancer Center is targeting 180 patients to compare chemo-PBT versus chemo-IMRT (NCT01512589) [48]. Importantly, in addition to examining progression-free survival and the so-called “total toxicity burden”, there are a number of highly relevant secondary endpoints which are also being evaluated. These include cost-effectiveness, patient-reported outcomes, and quality of life. These alternative endpoints will be imperative to characterizing the utility of PBT for EC from several clinically relevant angles. Importantly, they will pave the way for future investigations aiming to delineate the specific subpopulations that benefit most from PBT.

With the perspective of older trials all utilizing 3DCRT, it will be important to further address the utility of IMRT in EC. This is especially true because, whereas only a limited few facilities in the USA offer PBT, virtually all RT facilities in the USA are capable of IMRT. Though IMRT use in EC has certainly risen over time, a challenge in the future will be to provide high-quality evidence that IMRT is associated with fewer toxicities and/or postoperative complications over 3DCRT. Though it is unlikely that a phase III trial will be performed, the acceptance of the utility of IMRT for EC to be the standard of care will involve many more studies to demonstrate the benefit of IMRT. 

In summary, as medicine transitions into a new era of personalized care, both radiation oncology, and oncology as a whole, faces a multitude of challenges and unanswered questions, but also potentially paradigm-changing discoveries. Radiotherapy has progressed substantially during the past decade, and newly minted “standards of care” will likely undergo rapid continued evolution in the decades to come. A chief goal of these advancements will be to alleviate iatrogenic toxicities through increased precision at both the local and systemic levels. If successful, it is certainly possible that these “advancements” of precision can one day be hailed as the standard of care in multidisciplinary oncologic therapy.

## Figures and Tables

**Table 1:** Selected randomized trials examining neoadjuvant chemoradiation followed by surgery versus surgery alone.

Study	Groups	Chemotherapy	RT	Follow-up	Postoperative Complications	Mortality	L(R)R	Hospital Stay	Median OS
Walsh et al. * [9]	S (*n* = 55) vs. CRT + S (*n* = 58)	Cisplatin/5FU	40 Gy	0.8 years	Pulm: 58% vs. 48% Cardio: 24% vs. 24%	90 days: 4% vs. 9%	-	-	11 months vs. 16 months (*p* = 0.01)
Bosset et al. [15]	S (*n* = 139) vs. CRT + S (*n* = 143)	Cisplatin	18.5 + 18.5 split-course	4.6 years	General: 26% vs. 33% (*p* = 0.25)	Postoperative: 4% vs. 12% (*p* = 0.01)	RR 0.6, favoring CRT + S (*p* = 0.01)	24 days vs. 24 days (*p* > 0.05)	19 months vs. 19 months (*p* = 0.78)
Urba et al. [10]	S (*n* = 50) vs. CRT + S (*n* = 50)	Cisplatin/5FU/vinblastine	45 Gy	8 years	Wound/GI: 10% vs. 14% (*p* > 0.05)	Postoperative: 4% vs. 2% (*p* > 0.05)	42% vs. 19% (*p* = 0.02)	-	18 months vs. 17 months (*p* = 0.15)
Burmeister et al. [11]	S (*n* = 128) vs. CRT + S (*n* = 128)	Cisplatin/5FU	35 Gy	5.4 years	Pulm: 28% vs. 20% Cardio: 11% vs. 12% GI: 5% vs. 5%	Postoperative: 5% vs. 4% (*p* > 0.05)	19% vs. 15%	14 days vs. 14 days (*p* > 0.05)	19 months vs. 22 months (*p* = 0.57)
Tepper et al. * [12]	S (*n* = 26) vs. CRT + S (*n* = 30)	Cisplatin/5FU	45 Gy	6 years	Pulm: 54% vs. 54% Cardio: 13% vs. 4% GI: 29% vs. 29%	Postoperative: 4% vs. 0% (*p* > 0.05)	15% vs. 13%	10 days vs. 12 days (*p* > 0.05)	21 months vs. 54 months (*p* = 0.002)
van Hagen et al. [14]	S (*n* = 188) vs. CRT + S (*n* = 178)	Carboplatin/paclitaxel	41.4 Gy	3.8 years	Pulm: 44% vs. 46% Cardio: 17% vs. 21% GI: 30% vs. 22%	In-hospital: 4% vs. 4% (*p* = 0.70)	-	-	24 months vs. 49 months (*p* = 0.003)
Mariette et al. [16]	S (*n* = 97) vs. CRT + S (*n* = 98)	Cisplatin/5FU	45 Gy	7.8 years	Pulm: 53% vs. 40% Surg: 32% vs. 31% Infection: 11% vs. 18%	Postoperative: 3% vs. 11% (*p* = 0.05)	29% vs. 22% (*p* = 0.02)	15 days vs. 18 days (*p* = 0.80)	41 months vs. 32 months (*p* = 0.94)

RT, radiation therapy; L(R)R, loco(regional) recurrence; OS, overall survival; S, surgery; CRT, chemoradiotherapy; 5FU, 5-fluorouracil; Gy, Gray; RR, relative risk; GI, gastrointestinal (most commonly referring to anastomotic complications); * Denotes use of two-dimensional radiotherapy planning; remainder utilized three-dimensional conformal radiotherapy.

**Table 2:** Randomized evidence comparing chemoradiation and surgery.

Study	Groups	Chemotherapy	RT	Follow-up	Mortality	LC	Hospital Stay	Median OS
Chiu et al. [17]	CRT (*n* = 36) vs. S (*n* = 44)	5FU, cisplatin	50-60 Gy	1.5 years	Operative: 7%	44% vs. 41% (*p* = 0.77)	41 days vs. 27 days (*p* = 0.02)	21 months vs. 24 months (*p* = 0.34)
Stahl et al. [18]	IC + CRT (*n* = 86) vs. IC + CRT + S (*n* = 86)	IC: 5FU, VP16, cisplatin CRT: cisplatin, VP16	65 + Gy (no S), 40 Gy (with S)	6 years	Postoperative: 4% vs. 13% (*p* = 0.03)	43% vs. 62% (*p* < 0.05)	-	15 months vs. 16 months (*p* > 0.05)
Bedenne et al. [19]	CRT (*n* = 130) vs. CRT + S (*n* = 129)	5FU, cisplatin	46 Gy continuous or 15 + 15 Gy split-course	4 years	3 months: 1% vs. 9% (*p* = 0.002)	57% vs. 66% (*p* < 0.05)	52 days vs. 68 days (*p* = 0.02)	19 months vs. 18 months (*p* = 0.49)

RT, radiation therapy; LC, local control; OS, overall survival; CRT, chemoradiotherapy; S, surgery; 5FU, 5-fluorouracil; Gy, Gray; IC, induction chemotherapy; VP16, etoposide.

**Table 3:** Selected retrospective studies examining neoadjuvant intensity-modulated radiotherapy and chemotherapy followed by surgery.

Study	*N*	Chemotherapy	RT	Follow-up	Postoperative Complications	L(R)R	DM (+/− LR)	Median OS
La et al. [28]	30	Various	50.4 Gy	24 months	-	37%	40%	-
Wang et al. [26]	164	Various	50.4 Gy		Pulm: 24% Cardio: 17% GI: 18% Wound: 12% Death: 2% Hospital stay: 10 d	-	-	-
Shridhar et al. [29]	58	Cisplatin/5FU	50.4 Gy	19 months	Death: 5%	-	-	33 months
Freilich et al. [30]	138	Cisplatin/5FU	50.4 Gy	19 months	-	12%	26%	31 months
Zeng et al. [31]	17	Cisplatin/5FU	50.4 Gy; boost to 56 Gy	54 months	Surgical leak: 24%	11%	40%	29 months

*N*, sample size; RT, radiation therapy; L(R)R, loco(regional) recurrence; DM, distant metastasis; OS, overall survival; Gy, Gray; GI, gastrointestinal; 5FU, 5-fluorouracil.

**Table 4:** Selected retrospective studies examining concurrent proton beam therapy and chemotherapy.

Study	*N*	Chemotherapy	RT	Follow-up	Postoperative Complications	L(R)R	DM (+/− LR)	3-Year OS
Ishikawa et al. [42]	40	Cisplatin/5FU	60 GyE	24 months	-	34%	-	70%
Lin et al. [43]/Wang et al. [39]	62	Various	50.4 GyE	20 months	Pulm: 14% GI: 18% Death: 0% Hospital stay: 9 d	31%	26%	52%

*N*, sample size; RT, radiation therapy; L(R)R, loco(regional) recurrence; DM, distant metastasis; OS, overall survival; 5FU, 5-fluorouracil; GyE, Gray-equivalent; GI, gastrointestinal.
